# Home-based exercise program for adolescents with juvenile dermatomyositis quarantined during COVID-19 pandemic: a mixed methods study

**DOI:** 10.1186/s12969-021-00646-7

**Published:** 2021-11-13

**Authors:** Camilla Astley, Sofia Mendes Sieczkowska, Isabela Gouveia Marques, Bianca Pires Ihara, Livia Lindoso, Sofia Simão Martins Lavorato, Lucia Maria Arruda Campos, Rosa Maria Rodrigues Pereira, Adriana Maluf Elias, Nadia Emi Aikawa, Katia Kozu, Amanda Yuri Iraha, Tathiane Christine Franco, Hamilton Roschel, Ligia Bruni Queiroz, Guilherme Vanoni Polanczyk, Clovis Artur Silva, Bruno Gualano

**Affiliations:** 1 Applied Physiology and Nutrition Research Group, School of Physical Education and Sport, Universidade de São Paulo, São Paulo, Brazil; 2 Rheumatology Division, Hospital das Clínicas, Faculdade de Medicina, Universidade de São Paulo, HC-FMUSP, Av. Dr. Arnaldo, 455, 3° andar, São Paulo, SP 01246-903 Brazil; 3 Instituto da Criança e do Adolescente (ICr), Hospital das Clínicas, Faculdade de Medicina, Universidade de São Paulo, HC-FMUSP, São Paulo, Brazil; 4 Department of Psychiatry, Faculdade de Medicina, Universidade de São Paulo, São Paulo, SP Brazil; 5 Food Research Center, Universidade de São Paulo, São Paulo, Brazil

**Keywords:** Pediatric rheumatologic diseases, Lifestyle, Physical activity, Well-being, Myositis, COVID-19

## Abstract

**Background:**

Exercise has been suggested to prevent deterioration of health-related quality of life (HRQL) and overall health in pediatric rheumatologic diseases during the COVID-19 pandemic. Herein we describe the effects of a 12-week, home-based, exercise program on overall health and quality of life among quarantined patients with juvenile dermatomyositis (JDM).

**Method:**

This prospective, quasi-experimental, mixed methods (qualitative and quantitative) study was conducted between July and December 2020, during the most restricted period of COVID-19 pandemic in Brazil. The home-based exercise program consisted of a 12-week, three-times-a-week, aerobic and strengthening (bodyweight) training program. Qualitative data were systematically evaluated. Strengths and Difficulties Questionnaire (SDQ), Pediatric Quality of Life Inventory (PedsQOL) and Pittsburgh Sleep Quality Index (PSQI) evaluate symptoms of mental health disorder, HRQL, and quality of sleep.

**Findings:**

11 patients (out of 27) met the inclusion criteria (91% female; mean ± SD age: 13.5 ± 3.2 years). Adherence to the intervention was 72.6%. Barriers to exercise involved poor internet connectivity, excessive weekly sessions, and other commitments. Even though not statistically significant, Self-report SDQ subscales Total Difficulties Score, Emotional Problems Score, and PedsQOL School Functioning Score improved after intervention (− 2.4; 95%confidence interval [CI] -5.1; 0.2, *p* = 0.06; − 1.0; 95%CI -2.2; 0.2, *p* = 0.09 and; 11.7; 95%CI -2.5; 25.8, *p* = 0.09, respectively). Remaining SDQ subscales were not altered. Six themes emerged from patients’ and parents’ comments (qualitative results). Patients engaged in exercise reported other health-related benefits including increased motivation, concentration and strength.

**Interpretation:**

A home-based exercise program was associated with qualitative perceptions of improvements in overall health and HRQL by quarantined adolescents with JDM during COVID-19 pandemic. Lessons from this trial may help developing interventions focused on tackling physical inactivity in JDM.

## Highlights

1. This was the first study to explore the effects of exercise training in quarantined patients with JDM during the COVID-19 pandemic.

2. A home-based exercise training program was associated with borderline improvements in mental health disorder and quality of life. Patients engaged in exercise also reported other health-related benefits including increased motivation, concentration and strength.

3. Barriers to engage in the exercise program involved poor internet connectivity, excessive weekly sessions, and other commitments during quarantine, whereas facilitators included pleasure with exercise and framing exercise as fun. These data can provide support for the development of clinical exercise programs to patients with JDM, particularly during the COVID-19 pandemic.

## Introduction

Quarantine and social distance measures to mitigate coronavirus disease 2019 (COVID-19) pandemic have affected the physical and psychological well-being of children and adolescents [1]. Two recent surveys involving children and adolescents from Brazil demonstrated that school closures and home confinement due to the COVID-19 pandemic were responsible for reduction in daily physical activity, which directly impacts physical and mental health [2]. It has been suggested that children and adolescents are more likely to experience high rates of depression and anxiety symptoms during and after enforced isolation ends [2]. In the beginning of the pandemic, physicians believed that the scenario could be even worse for adolescents with rheumatic diseases as they may be at higher risk for developing severe COVID-19, therefore physicians also believed in that period that these patients needed more restrictive measures of social distancing. In thesis, this scenario could aggravate depression and anxiety symptoms and poor physical function and quality of life, which are common features among youth with pediatric rheumatologic diseases [3, 4]. However, children with rheumatic disease do not appear to present a higher risk of severe COVID-19 and outcomes of the SARS-CoV-2 infection in these patients are similar to those of general pediatric population [5–7].

Juvenile dermatomyositis (JDM) is a very rare childhood autoimmune condition characterized by inflammation of small vessels within the skin, muscle, and major organs. Prognosis is variable, ranging from monocyclic disease to chronic illness, and can extend into adulthood with possible complications [8]. Therapeutic exercises could improve physical function, aerobic conditioning, quality of life, and even disease activity among JDM patients [9]. Home-based exercise interventions have been suggested as a potentially safe and feasible tool to prevent deterioration of quality of life and overall health in confined patients with rheumatologic diseases, though data to support this hypothesis remain scarce.

Herein we describe the effects of a 12-week, home-based, exercise program on health-related quality of life, mental health, and sleep quality among quarantined adolescents with JMD during the COVID-19 pandemic.

## Methods

### Study design and patients

This was a prospective, quasi-experimental, mixed methods (quantitative and qualitative) study conducted between July 2020 and December 2020, during the COVID-19 pandemic. All patients stayed home and were on home-schooling throughout the study due to the outbreak. This study is part of a larger project aimed at exploring potential therapeutic effects of exercise during the COVID-19 pandemic in adolescents with a variety of chronic diseases (clinicaltrials.gov NCT04458246). Patients aged between 10 to 19 years were recruited from the Pediatric Rheumatology Unit of the Children’ and Adolescents’ Institute and the Juvenile Rheumatology Outpatient Clinic, Clinical Hospital, University of Sao Paulo (HC-FMUSP).

Inclusion criteria were 1) diagnosis with definite JDM according to the Bohan and Peter criteria [10], 2) receiving treatment or follow-up at HC-FMUSP, 3) age between 10 and 19 years. Absence of i) cardiovascular involvement (e.g., arrhythmias, arterial hypertension, heart failure, conduction disturbances, myocarditis, or pericarditis), ii) undernourishment, or iii) chronic kidney disease or iv) chronic pulmonary disease. All patients obtained medical clearance to participate in the intervention. The patients were not engaged in any form of exercise for at least 3 months prior to and during the study.

The Research Electronic Data Capture (REDCap®) was used to send questionnaires to patients, before and after the intervention. REDCap® is a safe web tool planned to support data capture for research studies. It also audits trails for tracking data manipulation and allows automated data export procedures for statistical analyses. Up to six emails or messages were sent to improve the response rate. Qualitative data were also collected during and after the intervention (see details below). The study was approved by the National Ethics Committee (CONEP number 4.081.961) and the patients’ parents or guardians signed an online consent prior to participate in the study.

### Home-based exercise program

The home-based exercise program consisted of a 12-week aerobic and bodyweight exercise training program [11]. Training sessions were divided into two parts. Initially, the warm-up included predominantly aerobic exercises such as jumping jacks, skipping, and mobility and flexibility exercises. The second part included bodyweight exercises for the major muscle groups such as squats, lunges, push-ups, crunches, and planks. Exercise sessions occurred three times a week. One weekly session was conducted with online live supervision with the trainer, whereas the other two weekly sessions were unsupervised, but patients were instructed to provide feedback to the trainer immediately after completion of the training session. Supervision/monitoring was conducted via WhatsApp® or Google Meets® according to patients’ preference. Progression occurred every 4 weeks by increasing the number of sets (3 to 4), repetitions (10 to 15) and/or duration (30 to 45 s). Sessions included 1 to 5 patients at a time and adherence to the exercise program was monitored on a session basis by a member of the research staff.

Patients received instructional videos, photos, and “gifs” describing and illustrating the exercise program. Before the commencement of the training program, a video call was conducted with all patients (and their parents, whenever necessary), individually, in order to provide all of the details necessary to perform the exercise training program and to collect information on patient’s health status.

Adherence to the training program was assessed by means of training log. Supervised sessions had immediate assurance of adherence by the trainer, whereas adherence to the unsupervised sessions was assessed via feedback provided by the patients immediately after completion of the training session at home.

### Strengths and difficulties questionnaire

The Strengths and Difficulties Questionnaire (SDQ) was used to evaluate symptoms of mental health disorder. It is a behavioural screening tool used to assess social, emotional, and physical aspects of behaviour in young people [12] that has been shown to be valid and reliable [13]. The questionnaire has 25 items which comprise five sub-scales: (i) emotional symptoms (anxiety and depressive symptoms); (ii) conduct problems; (iii) hyperactivity/inattention; (iv) peer relationship problems; and (v) prosocial behaviour (positive behaviours such as being kind and helpful, scored in reverse of the other subscales). Response options are ‘not true, somewhat true, or certainly true’ (scored 0, 1 or 2). The SDQ Total Difficulties Score (SDQ TDS) was generated by adding together the scores from the first four subscales ranging from 0 (low difficulties) to 40 (high difficulties). The five sub-scales, with scores varying from 0 to 10, were also investigated independently. SDQ TDS provides a useful indicator of the level of overall symptoms of mental health disorder and is validated to Portuguese language [14]. In addition, the subscale items may be used to indicate specific clinical disorders in adolescents: depression, anxiety, hyperactivity attention deficit disorder (ADHD) and behavioural/conduct disorder.

### Pediatric quality of life inventory – PedsQL 4.0 generic Core scale

The PedsQL 4.0 [15] is widely used in other countries and is validated to Portuguese langue [16] ref). It focuses on the following domains: physical (eight items), emotional (five items), social (five items), and school (five items). The PedsQL is composed by child self-report and parent proxy-report. All items for each of the forms are essentially identical, differing only in language. The answers are rated on a five-point scale (0 = never, 1 = almost never, 2 = sometimes, 3 = often, 4 = almost always). The items are inversely scored and transposed on a 0 to 100 scale (0 = 100, 1 = 75, 2 = 50, 3 = 25, 4 = 0). Thus, the greater the score, the higher the quality of life. The total score is a sum of the scores across the four dimensions evaluated. The physical summary corresponds to the mean of the physical dimension (eight items), while the psychosocial summary (15 items) covers the emotional, social, and school domains.

### Pittsburgh sleep quality index (PSQI)

Sleep quality was assessed by PSQI [17, 18]. The questionnaire is composed by 19 questions representing one of the seven components of sleep quality: subjective sleep quality, sleep latency, sleep duration, sleep efficiency, sleep disturbance, sleep medication intake, and daytime dysfunction. Each component score was rated on a 3-point scale, leading to a sum of up to 21 points. A PSQI score > 5 indicated a poor sleep quality whereas a PSQI score ≤ 5 indicated a good sleep quality.

### JDM scores

JDM scores parameters included the Manual Muscle Test (MMT) [19], Childhood Muscle Assessment Scale (CMAS) [20], Disease Activity Score (DAS) [20].

### Qualitative analysis

Qualitative data were collected at two timepoints: 4 weeks into the exercise program, and after the program completion. The 4-week data were collected during a videocall with the trainer, and program completion feedback was collected via text or voice message sent directly to the trainer. Data was transcribed verbatim in Portuguese and translated to English by bilingual and bicultural researchers. Themes were derived from the data using content analysis [21]. In this two time points, the following questions were asked: i) What did you like about the exercise training program?; ii) What did you not like about the exercise training program?; iii) How could the exercise training program be improved and what could have been done different to stimulate your engagement?; iv) Did you feel any difference in your general health due to the exercise program?

### Statistical analysis

Wilcoxon tests were used to assess possible differences between pre- and post-intervention in a complete case analysis. Significance level was previously set at *p* ≤ 0.05. Data are expressed as mean ± SD, delta changes and 95% confidence interval (95%CI).

## Results

### Participants’ characteristics

**Fig. 1:**
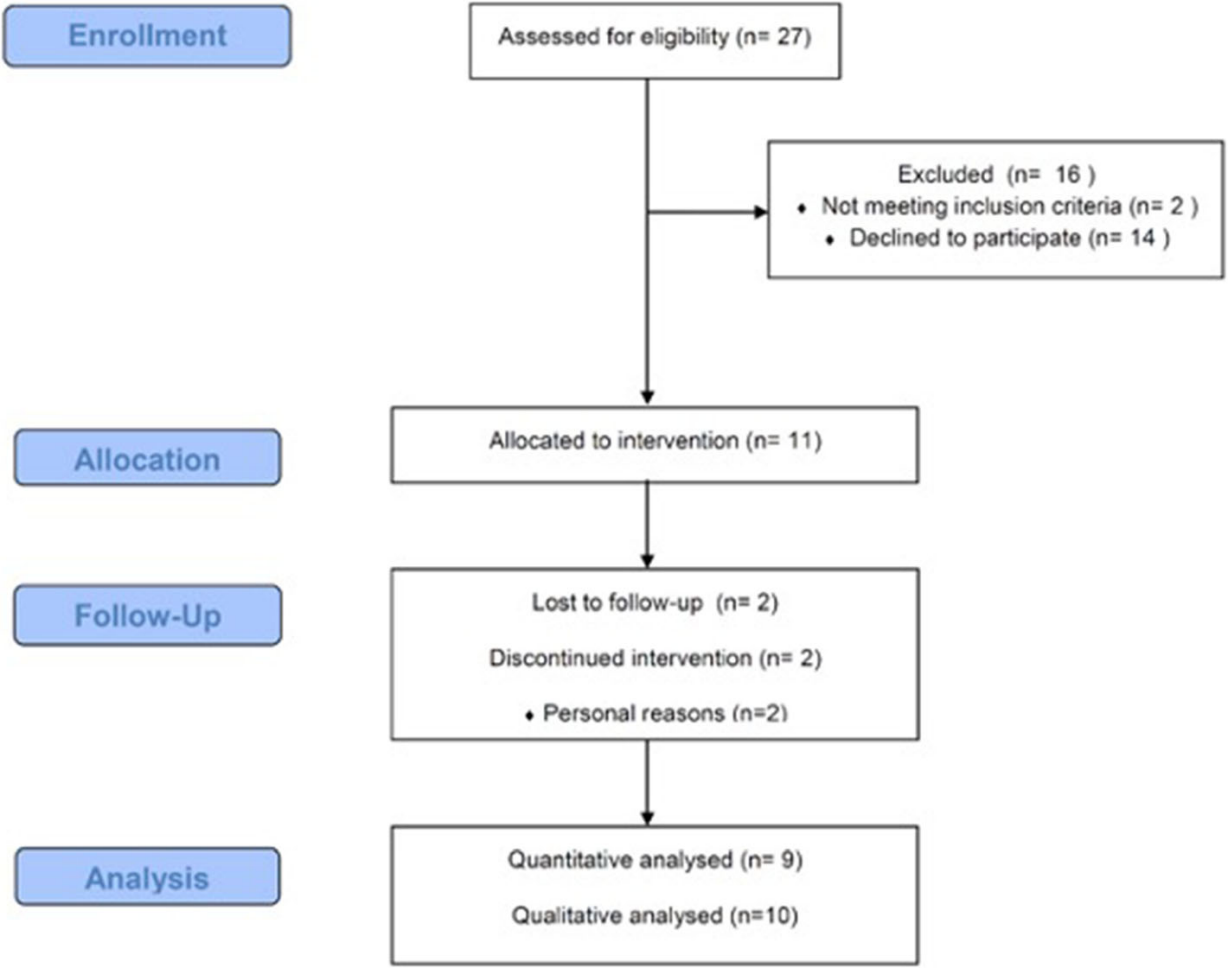
Flow chart of patients

Among 27 patients assessed for eligibility, two patients were unable to participate due to physical limitations and 14 declined to participate. Thus, 11 met the inclusion criteria and took part in this study (91% female; age: 13.5 ± 3.2 years; Body Mass Index (BMI): 21.7 ± 6.6 kg/m^2^; time elapsed since diagnosis: 7.6 ± 4.6 years). During the follow-up period, 2 patients dropped out due to personal reasons (Fig. 1), but their data were kept in the qualitative analysis. Three patients were currently using prednisone (median 0.26 mg/kg/day), 4 were using methotrexate and 5 hydroxychloroquine (Table 1). Adherence to the exercise protocol was 72.6% (dropouts included) and 80.9% (excluding dropouts). Importantly, there were no partially completed training sessions and there were no adverse effects reported by the patients.

**Table 1:** Patients’ demographic characteristics, drug regimen and adherence to the protocol

Patient	I	II	III	IV	V	VI	VII	VIII	IX	X	XI
Sex	F	M	F	F	F	F	F	F	F	F	F
Age (years)	10	10	11	11	12	12	14	16	17	19	18
BMI (kg/m^2^)	15.5	20.8	15.4	27.8	20.1	20.6	21.6	21.2	38.6	21.8	16.2
BMI percentile	25th	97th	15th	99th	85th	85th	75th	50th	99th	50th	3th
Duration of disease (years)	7	7	4	2	5	7	10	2	13	17	10
Prednisone dose (mg/kg/day)	–	–	–	0.16	0.11	–	–	0.51	–	–	–
Current use of other rheumatic drugs	HCQ, MTX	–	HCQ	HCQ, MTX	MTX	–	–	HCQ, MTX	–	–	HCQ
CMAS (0–52	52	52	52	52	NA	52	NA	52	52	NA	50
MMT (0–80)	80	80	80	80	NA	80	NA	80	80	NA	80
DAS (0–28)	0	0	4	0	NA	4	NA	0	3	NA	0
Adherence (%)	96.7	93.3	66.7	10.0	80.0	73.3	100	16.7	66.7	73.3	83.3

*BMI body mass index, *MTX* methotrexate, *HCQ* hydroxychloroquine. BMI percentile classification: underweight: < Percentile 3; normal: ≥ Percentile 3 and < Percentile 85; overweight: ≥ Percentile 85 e < Percentile 97; obese: ≥ Percentile 97. CMAS: Childhood Muscle Assessment Scale; MMT: Manual Muscle Test; DAS: Disease Activity Score

### Quantitative results

The SDQ TDS, SDQ domains and PedsQL domains and PSQI scores did not change after the intervention (Table 2).

**Table 2:** Effects of a 12-week home-based exercise training program on Strengths and Difficulties Questionnaire (SDQ), Pediatric Quality of Life Inventory 4.0 (PedsQL 4.0) and Pittsburgh Sleep Quality Index (PSQI) in patients with juvenile dermatomyositis

Domains (score)	Baseline	Post intervention	95%CI	***P***
**SDQ**	**(*n*** **= 9)**	**(*n*** **= 9)**		
**Total difficulties score (0–40)**	11.67 (6.46)	9.22 (6.49)	−2.44 (−5.08; 0.19)	0.05
**Peer problems (0–10)**	1.44 (1.59)	1.11 (0.92)	−0.33 (−1.42;0.75)	0.38
**Emotional problems (0–10)**	3.56 (2.40)	2.56 (2.06)	−1.00 (−2.21;0.21)	0.08
Internalization scale (0–20)	5.00 (3.31)	3.66 (2.59)	−1.33 (−3.21;0.54)	0.14
**Conduct problems (0–10)**	2.67 (2.29)	2.00 (1.93)	−0.66 (−2.29;0.96)	0.32
**Hyperactivity/inattention (0–10)**	4.00 (3.24)	3.56 (3.67)	−0.44 (−1.83;0.94)	0.54
Externalization scale (0–20)	6.66 (4.71)	5.55 (5.29)	−1.11 (−3.46;1.24)	0.30
**Prosocial (0–10)**	8.67 (1.50)	7.89 (2.42)	−0.77 (−2.48;0.93)	0.35
**Impact score (0–10)**	1.22 (1.71)	1.22 (1.92)	−0.00 (− 0.38;0.38)	1.00
**PedsQL**	**(*n =*** **9)**	**(*n =*** **9)**		
**PedsQL 4.0 total scale score (0–100)**	73.78 (16.27)	77.14 (16.26)	3.35 (−4.93;11.64)	0.55
**Physical health summary score (0–100)**	75.34 (19.3)	77.43 (22.95)	2.08 (−7.89;12.06)	0.62
**Psychosocial health summary (0–100)**	72.22 (14.36)	76.85 (12.03)	4.62 (−3.54;12.80)	0.28
Emotional functioning (0–100)	67.78 (12.52)	68.89 (15.36)	1.11 (−9.89;12.11)	0.83
Social functioning (0–100)	86.11 (16.91)	87.22 (17.87)	1.11 (−6.99 to 9.21)	0.73
School functioning (0–100)	62.78 (26.58)	74.44 (16.47)	11.66 (−2.45; 25.78)	0.05
**PSQI**	**(*n =*** **9)**	**(*n =*** **9)**		
**PSQI total score (0–21)**	5.89 (2.61)	5.11 (3.25)	−0.78 (−3.54;1.98)	0.52
Overall sleep quality (0–3)	0.67 (0.70)	0.78 (0.66)	0.11 (−0.48;0.71)	0.65
Sleep latency (0–3)	1.44 (1.13)	1.0 (1.22)	−0.44 (−1,12;0.23)	0.15
Sleep duration (0–3)	0.11 (0.33)	0.0 (0)	−0.11 (− 0.36;0.14)	0.31
Sleep efficiency (0–3)	0.56 (1.01)	0.33 (0.70)	−0.23 (−1.06; 0.61)	0.65
Sleep disturbances (0–3)	0.89 (0.33)	1.0 (0.50)	0.11 (−0.35;0.57)	0.56
Sleep medication use (0–3)	1.22 (1.48)	1.11 (1.45)	−0.11 (−1.57;1.34)	0.89
Daytime dysfunction (0–3)	1.0 (0.70)	0.89 (0.60)	−0.11 (− 0.57;0.35)	0.56

*Results are presented in median (SD). *SDQ* Strengths and Difficulties Questionnaire, *PedsQL 4.0* Pediatric Quality of Life Inventory and *PSQI* Pittsburgh Sleep Quality Index.

### Qualitative results

Six themes emerged from patients’ and parents’ comments (Table 3): 1) suitability of the home-based format; 2) appropriate trainer supervision; 3) appropriate exercises; 4) online group sessions as a motivating factor; 5) online group sessions as a barrier, and 6) health benefits.

**Table 3:** Qualitative analyses after 12-wk home-based exercise program in JDM patients

Themes	Patient’s	Quotes
Suitability of the home-based format	II	*“I liked the online format because it helped me (…*), *because the trainer can oversee the training and see how I am doing even distant, and I had no problems with the internet”*
	VII	*“I enjoyed having an online consultancy and the progression every other week”. “I really liked it, because it was something complete and technological”*
	IX	*“I felt very comfortable and at ease with the form of training, with the trainer, and how the contact was made.”*
	X	*“I liked a lot that the program was online and I could exercise at my own schedule”*
	XI	*“I think the whole training program pleased me, the online format worked very well, exceeding my expectations”*
Appropriate trainer supervision	I	*“I enjoyed having an [online] session with the trainer, I think she explains the exercises well”*
	II	*“I liked the trainer”*
	III	*“I liked the way the trainer taught, it was easy to understand”*
	V	*“I liked to exercise with the trainer “*
	VI	*“The trainer it is great, [she] is patient.”*
	VII	*“The training motivated me, because there was a trainer always motivating me, sending a message to know how I was doing … It was an elaborated program. And that helped me a lot to continue”*
	VIII	*“I liked the trainer, patient and calm”*
	IX	*“I really enjoyed training with the trainer.”*
	X	*“I liked the trainer and her way of conducting the training [session].”*
	XI	*“The trainer was excellent and super attentive. In the end the exercises were all to my liking, always following the level of difficulty”*
Appropriate exercises	I	*“I liked the type of exercises”*
	II	*“I really enjoyed the exercise of running in the same place”*
	III	*“I liked the exercises; I used to do them before. I would prefer to play fun games like dodgeball, but I know we can’t do it online.”*
	VI	*“I liked the exercises, but some exercises were difficult”*
	VII	*I liked the exercises, jumping jacks, squats, sit-ups* etc.
	IX	*I liked that the exercises are done at home. The exercises are light, there is no need of equipment, and they are not super difficult. The exercises are also adaptable.*
	X	*“I really enjoyed the exercises”*
	XI	*“The exercises were all to my pleasing. There were some difficult and tiring ones, but I really enjoyed doing them, in general, I thought I managed to do them all. They were good exercises.*”
Motivators and facilitators to the program	I	*“It was good, because in a group one can see new people, and I feel more encouraged to do the exercise in a group.”*
	VII	*“I liked it, because I can watch others doing the exercises that I can’t”*
	IX	*“The coolest thing about the program is doing it at home.”*
	X	*“I enjoyed the group classes; I had fun with the classes.”*
	XI	*“I was missing being active. Before the pandemic I was more active in terms of routine of physical activity, and not exercising for a long time bothers me”. “I liked it [online group sessions]. At first, I was shy but then I saw that everyone had difficulties and I did not feel bad because at some exercises I did well, but not at other exercises. But my peers were just like me”*
Barriers of the program	I	*“The internet got in the way a little bit, sometimes it got frozen”*
	II	*“I did not like some of the exercises, that were too difficult.”*
	V	*“I do not like to do squats”, “I did not like the types of exercise”, “I did not like the group sessions. Because I could not focus. And also, I did not like the frequency of the online sessions, most of the times I had to stop something that I was doing to participate in the online session”*
	VI	*“I did not like the online format”*
	VIII	*“My routine was incompatible, I had to work and study”*
Health benefits	I	*“It made me stronger, sleep improved, I was more energetic, less tired in everyday activities.”*
	II	*“I felt more spirited, cheerful”*
	III	*“My sleep improved”*
	V	*“Concentration”*
	VI	*“I felt more energetic my concentration has improved.”*
	VII	*I felt more energetic, less tired*
	IX	*“I felt less pain and fatigue”. “I felt more motivate and cheerful in the days that I exercise”*
	X	*“I found that my mood and sleep improved a lot.”*
	XI	*“I felt differences such as more disposition and better nights of sleep. Regarding pain, the muscles used in the exercises were always sore the next day, but nothing abnormal and then I always got used to it and got more tolerant.”*

#### Suitability of the home-based format

Patients reported that the home-based exercise program was generally suitable to be performed during the COVID-19 pandemic. Patients and parents cited that the flexibility to exercise at a time that best suits patients’ schedule was very helpful to maintain adherence to the program. One patient acknowledged that the support provided at an in-person exercise training is greater than in a home-based format; however, she said it was comfortable to exercise at home.

“I liked a lot that the program was online and I could exercise at my own schedule” – Female patient, 19 years old.

#### Appropriate trainer supervision

All patients reported great satisfaction with the interaction with the exercise trainers. Patients stated that the trainers were very helpful with motivating to exercise and checking in regarding barriers and difficulties to adhere to the program. Patients described the exercise trainers as supportive, charismatic, attentive, and friendly. Patients cited that the trainer assisted with the exercise movement and also by providing social support. All patients except one were satisfied with once-a-week online supervised sessions.

“The training motivated me, because there was a trainer always motivating me, sending a message to know how I was doing, there was something more elaborate. And that helped me a lot to continue” – Female patient, 14 years old.

#### Appropriate exercises

The majority of patients enjoyed the exercises. A few exercises were less preferred such as squatting and sit-ups. Patients reported they felt somewhat tired during the exercise sessions; however, they were not exhausted after the training session. They also mentioned they felt more tired after each training progression; nevertheless, they got used to the exercises towards the end of progression level and the exercises became easier. The youngest patient (10 years old) stated she would prefer to engage in fun or group activities; however, she understands it would not be able to be instructed online.

“I liked that the exercises are done at home, that they are light, no equipment is needed, exercises are not difficult, and they are easy to be adapted” – Female patient, 17 years old.

#### Motivators and facilitators to the program

The online group sessions were pleasant for some participants. Having their peers in a group session assisted with accountability and facilitated adherence to the program. Some patients said they enjoyed observing how other patients also faced difficulties to perform complex movements. For some patients exercising at home was very interesting and innovative. One patient said that the training and the trainer motivate her. The group sessions were perceived as fun for some patients, and they are glad to join it.

“I liked it [online group sessions]. At first, I was shy but then I saw that everyone had difficulties and I did not feel bad again because at some exercises I did well, but not at other exercises. But my peers were just like me” – Female patient, 18 years old.

#### Barriers to the program

The online group sessions were perceived by some patients as a negative aspect of the program. Some patients reported feeling shy during the sessions. Another patient stated to prefer individual sessions because conversation distracted her from exercising. One patient reported poor internet connectivity during the online group sessions, which interfered with the quality of the session. One patient cited that online group sessions were too frequent and she had to reschedule some of her commitments to attend the sessions. Some patients reported that they did not like some exercises. And one patient reported that she could not continue to participate because she had to work and study.

“I did not like the group sessions, because I could not focus. And also did not liked the frequency of the online sessions, most of the times I had to stop something that I was doing to participate in the online session” – Female patient, 12 years old.

#### Health benefits

All participants reported improvements in health after completing the program. The most cited health benefit was better mood. Patients also reported better sleep quality, motivation, concentration and strength. Reduced disease-related symptoms were also mentioned.

“I felt less pain and fatigue … I felt more motivate and cheerful in the days that I exercise” – Female patient, 17 years old.

## Discussion

To our knowledge, this was the first study to explore the effects of exercise training in quarantined adolescents with JDM during the COVID-19 pandemic. The main findings suggest that a home-based exercise training program was associated with borderline improvements in mental health disorders and quality of life. Patients engaged in exercise also reported other health-related benefits, including increased motivation, concentration and strength. Barriers to the exercise were heterogeneous and involved poor internet connectivity, excessive weekly sessions, and other commitments (e.g., work and study) during quarantine, whereas facilitators included pleasure with exercise and framing exercise as fun. These data can inform the development of clinical exercise programs to patients with JDM, particularly during the COVID-19 outbreak.

Patients with JDM generally present with muscle weakness, poor aerobic conditioning and exercise intolerance [22–24]. These characteristics have been associated with increased systemic and capillary inflammation in muscle, low muscle mass and chronic use of glucocorticoid [22, 23, 25]. In addition, hypoactivity is a common feature in this disease. In an assessment of 19 consecutive patients with JDM, we found that only two achieved the physical activity guidelines [26]. We and others [27–29] have proposed that physical inactivity is a major factor leading to overall health deterioration in pediatric rheumatic diseases, with exercise being a therapeutic tool to offset the detrimental effects of inactivity. In fact, preliminary evidence has suggested that an exercise training could be effective in improving physical function and quality of life in JDM, without exacerbating the disease [30, 31]. As the social distancing measures due to the pandemic have led to reductions in physical activity levels among healthy individuals and patients with chronic diseases [32–34], we speculated that a home-based exercise program could benefit patients with JDM during the outbreak.

In general, the adherence to the program was satisfactory, and the mixed methods analysis revealed some benefits in overall health and well-being associated with exercise. Nonetheless, data related to mental health (SDQ) and quality of life (PedsQL) were not consistently improved, possibly as a reflection of the high interindividual variability. In fact, in the qualitative analysis, some patients reported improvements in fatigue, quality of sleep, pain and strength, while others found it difficult to follow the online program due to various reasons, such as feeling shy during the sessions, facing problems with internet connectivity, and disliking some exercises. These findings ultimately suggest that the feasibility of the intervention was partial, which may have influenced the extent of the beneficial effects experienced by the patients.

There is a paucity of studies assessing the effects of exercise training intervention in JDM. In a prospective, non-controlled trial involving JDM patients (*n* = 10), a supervised, 12-week, aerobic plus resistance training program led to improvements in peak oxygen consumption, time to exhaustion, muscle strength and function, increases in bone mineral apparent density, health-related quality of life, and reduced disease activity score [25]. The beneficial effects of exercise were subsequently extended to JDM patients (*n* = 9) who had recovered from disease flare. In this study, a 12-week aerobic training program resulted in improvements in maximal and submaximal aerobic capacity, with no evidence of muscle damage [25]. As the patients in the current study were deprived of in-hospital medical care due to the restrictions imposed by the pandemic, we were unable to assess clinical and biochemical outcomes as well as objective measures of physical function and capacity, which hampers direct comparisons with previous studies. To our knowledge, there is only one single study assessing the effects of a home-based exercise training program in JDM patients (*n* = 26) [31]. Following a 12-week intervention comprising interval training on a treadmill and strength exercises, patients who exercised showed greater improvements vs. controls in standing long jump distance, push-up and sit-up performance, and physical function, although isometric muscle strength and perception of fatigue remained unaltered [31]. The current data reinforce the utility of home-based exercise as a tool to maintain or increase physical activity in JDM, thereby preserving patients’ overall health and quality of life, which is particularly relevant during the COVID-19 pandemic, a period associated with increased physical inactivity and sedentariness.

The strengths of this study involved the investigation of patients with a rare condition deprived of face-to-face health care during the pandemic; the use of a mixed methods approach that provides broad assessment of the intervention; and the development of a novel home-based exercise training program tailored to pediatric patients with chronic conditions. The limitations included the small sample size; the absence of control group, which precludes establishing cause-and-effect relationships; and the lack of more objective outcomes and post-intervention clinical assessments, which was not possible because in-hospital care was temporarily suspended due to the pandemic.

## Conclusion

In conclusion, a home-based exercise program was associated with qualitative perceptions of improvements in overall health and HRQL by a small cohort of quarantined adolescents with JDM during COVID-19 pandemic. Lessons learned from this trial may help develop cross-culturally adapted, exercise interventions focused on tackling physical inactivity in JDM, especially during these unprecedented times when patients have stayed home to avoid infection complications.

## Data Availability

Access to de-identified data or related documents can be requested through submission of a proposal with a valuable research question, necessary data protection plan, and ethical approvals. A contract will be signed. Data requests should be addressed to the corresponding author.

## Abbreviations

COVID-19: Coronavirus disease 2019
JDM: Juvenile dermatomyositis
SDQ: Strengths and Difficulties Questionnaire
SDQ TDS: Total Difficulties Score
PedsQL: Pediatric Quality of Life Inventory
PSQI: Pittsburgh Sleep Quality Index
